# Coral mucus fuels the sponge loop in warm- and cold-water coral reef ecosystems

**DOI:** 10.1038/srep18715

**Published:** 2016-01-07

**Authors:** Laura Rix, Jasper M. de Goeij, Christina E. Mueller, Ulrich Struck, Jack J. Middelburg, Fleur C. van Duyl, Fuad A. Al-Horani, Christian Wild, Malik S. Naumann, Dick van Oevelen

**Affiliations:** 1 Coral Reef Ecology Group (CORE), Leibniz Center for Tropical Marine Ecology (ZMT), Fahrenheitstr. 6, 28359 Bremen, Germany; 2 Department of Aquatic Environmental Ecology, Institute for Biodiversity and Ecosystem Dynamics, University of Amsterdam, PO Box 94248, 1090 GE Amsterdam, the Netherlands; 3 Royal Netherlands Institute for Sea Research (NIOZ-Yerseke), PO Box 140, 4400 AC Yerseke, the Netherlands; 4 Museum für Naturkunde, Leibniz Institute for Evolution and Biodiversity Science, Invalidenstr. 43, 10115 Berlin, Germany; 5 Department of Earth Sciences – Geochemistry, Utrecht University, PO Box 80.021, 3508 TA Utrecht, the Netherlands; 6 Royal Netherlands Institute for Sea Research (NIOZ-Texel), PO Box 59, 1790 AB Den Burg, Texel, the Netherlands; 7 University of Jordan – Aqaba and Marine Science Station (MSS), PO Box 2595, Aqaba 77110, Jordan; 8 Faculty of Biology and Chemistry (FB 2), University of Bremen, NW 2/Leobener Str., 28359 Bremen, Germany

## Abstract

Shallow warm-water and deep-sea cold-water corals engineer the coral reef framework and fertilize reef communities by releasing coral mucus, a source of reef dissolved organic matter (DOM). By transforming DOM into particulate detritus, sponges play a key role in transferring the energy and nutrients in DOM to higher trophic levels on Caribbean reefs via the so-called sponge loop. Coral mucus may be a major DOM source for the sponge loop, but mucus uptake by sponges has not been demonstrated. Here we used laboratory stable isotope tracer experiments to show the transfer of coral mucus into the bulk tissue and phospholipid fatty acids of the warm-water sponge *Mycale fistulifera* and cold-water sponge *Hymedesmia coriacea*, demonstrating a direct trophic link between corals and reef sponges. Furthermore, 21–40% of the mucus carbon and 32–39% of the nitrogen assimilated by the sponges was subsequently released as detritus, confirming a sponge loop on Red Sea warm-water and north Atlantic cold-water coral reefs. The presence of a sponge loop in two vastly different reef environments suggests it is a ubiquitous feature of reef ecosystems contributing to the high biogeochemical cycling that may enable coral reefs to thrive in nutrient-limited (warm-water) and energy-limited (cold-water) environments.

Scleractinian corals act as ecosystem engineers on warm-water (WW) and cold-water (CW) coral reefs by forming the complex 3D-reef framework and driving reef biogeochemical cycles^1^,^2^. While WW coral reefs thrive in the warm, shallow and oligotrophic waters of the tropics, CW reefs are globally distributed along continental shelves, slopes and seamounts in the cold, deep, nutrient-rich waters below the photic zone^3^. On shallow WW reefs, corals form endosymbiotic associations with photosynthetic dinoflagellates (zooxanthellae), enabling them to contribute to high benthic autotrophic productivity^4^,^5^. Deep-sea CW corals by contrast lack zooxanthellae and instead rely on heterotrophic feeding to meet their energetic requirements^6^,^7^,^8^. Inorganic nutrient availability may limit the autotrophic primary productivity of WW coral reefs in oligotrophic waters^5^,^9^,^10^. Conversely, the metabolism of CW reefs relies on secondary production, and therefore is limited by the quantity and quality of the external organic carbon (C) input from the surface ocean via benthic-pelagic coupling^3^,^11^. Consequently, CW reefs are typically restricted to oceanic regions with high surface primary production and enhanced vertical transport due to elevated currents^12^,^13^,^14^. Despite these pronounced environmental differences (see Supplementary Table S1) both WW and CW reefs are considered hotspots of marine biodiversity and biological activity^3^,^5^,^15^. The mechanisms by which reefs deal with inorganic nutrient (WW) and organic C (CW) limitation are under debate, but efficient pathways of energy and nutrient cycling are essential for maintaining the high productivity of these contrasting reef ecosystems. Here, we propose that a trophic link between two reef ecosystem engineers, scleractinian corals and sponges, contributes to sustaining WW and CW reef ecosystems through the recycling of coral mucus, a key organic resource on shallow and deep-sea coral reefs^16^,^17^,^18^.

Scleractinian corals secrete a surface mucus layer that is continuously released into the water column in particulate and dissolved forms, thereby substantially contributing to reef organic matter pools^16^,^17^,^19^. Despite environmental and metabolic differences, WW and CW corals release mucus at comparable rates^17^,^20^,^21^ and devote substantial energy into mucus production; up to 40% of the net C fixed by WW corals^8^,^22^,^23^. Composed of a complex mixture of carbohydrates, lipids, and proteins, coral mucus is an energy-rich substrate that acts as an important energy and nutrient carrier on coral reefs^16^,^24^. The particulate fraction of released mucus functions as a particle trap^16^ facilitating the formation of aggregates that can act as substrate for various reef organisms^24^. However, the majority of released mucus (56–80%) dissolves in surrounding reef waters^16^,^17^, making it largely unavailable for most reef fauna. Research on mucus recycling has primarily focused on microbial degradation in the water column and reef sediments, where it rapidly stimulates bacterial growth and respiration enabling remineralization and recycling via the microbial loop^18^,^25^,^26^.

Recently, the so-called “sponge loop” has been identified as an alternative pathway for transferring dissolved organic matter (DOM) to higher trophic levels on Caribbean WW reefs^27^. Despite largely being considered particle feeders^28^,^29^, an increasing number of sponge species have been found to feed on DOM, with DOM accounting for up to 90% of the sponge diet^30^,^31^,^32^,^33^,^34^. Sponges subsequently transform a substantial fraction of this DOM into particulate organic matter (POM) via detritus production, effectively turning over up to 35% of their body C per day^27^,^35^,^36^,^37^. Sponge detritus is fed on by a variety of benthic detritivores, enabling the energy bound in DOM—that is otherwise unavailable to most reef heterotrophs—to be utilized by higher trophic levels^27^. In the Caribbean, DOM turnover through sponges amounts to the same order of magnitude as the total gross primary production rates of the entire reef ecosystem^27^. The sponge loop therefore plays a major role in organic matter cycling on Caribbean reefs, but has not yet been investigated in other oceanic regions. Moreover, de Goeij *et al.*^27^ only showed recycling of laboratory-produced diatom DOM within the sponge loop, which may not be representative of natural reef DOM. Since coral mucus contributes to reef DOM pools, the sponge loop may play a role in its recycling, but uptake of coral mucus by reef sponges has not been demonstrated.

Here, we used stable isotope tracer experiments with ^13^C- and ^15^N-enriched coral mucus as the “tracer” to investigate coral mucus recycling by the sponge loop (methods summarized in Supplementary Fig. S1). Laboratory flow-through and incubation set-ups were conducted to first trace the uptake of naturally produced coral mucus from ^13^C- and ^15^N-labeled corals (WW: Fungiidae; CW: *Lophelia pertusa*) into the sponges *Mycale (Carmia) fistulifera* (WW) and *Hymedesmia (Stylopus) coriacea* (CW). Additional incubations demonstrated the transfer of coral mucus into the sponge-produced detritus. Assimilation of coral mucus C into sponge phospholipid-derived fatty acids (PLFAs) was measured to further examine the processing of coral mucus by the sponges and evaluate the potential role of sponge-associated bacteria in its uptake.

## Results

### Coral mucus labelling

**Figure 1:**
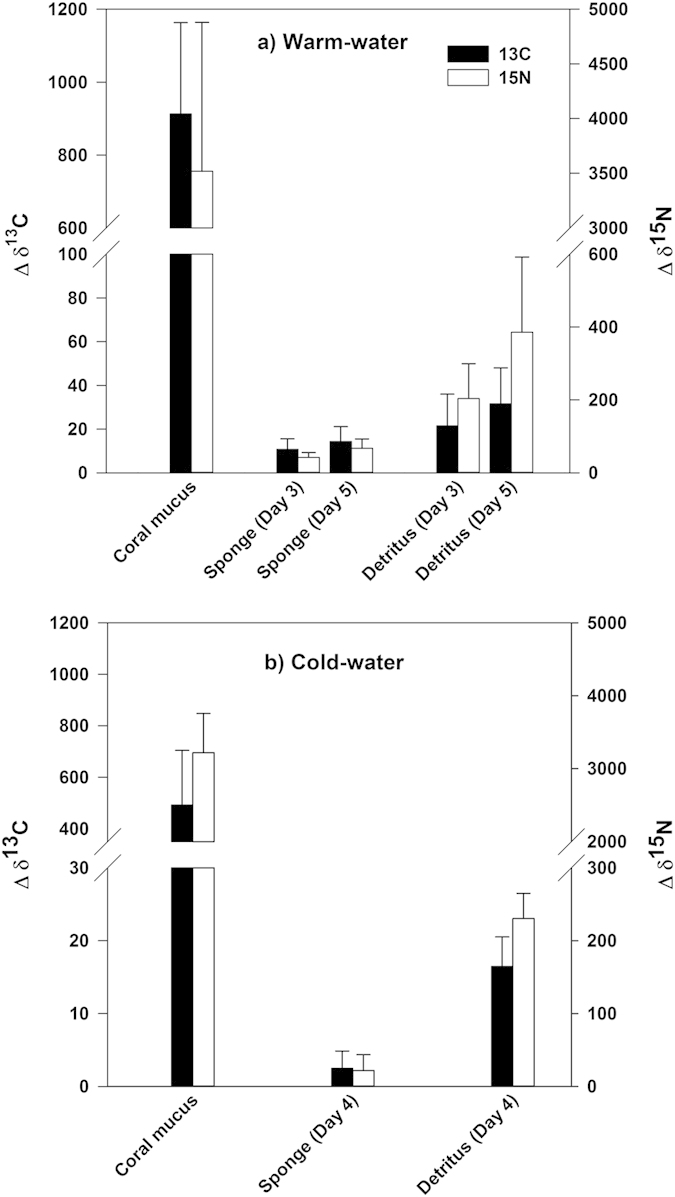
Stable isotope enrichment of ^13^C and ^15^N in coral mucus, sponge tissue and sponge detritus. Above-background isotope tracer incorporation Δδ^13^C (%) (dark bars) and Δδ^15^N (%) (light bars) in: (**a**) coral mucus, sponge tissue, and sponge detritus from the warm-water sponge *Mycale fistulifera*; with tissue and detritus sampled after 3 and 5 days exposure to ^13^C and ^15^N labeled warm-water coral mucus, and (**b**) coral mucus, sponge tissue, and sponge detritus from the cold-water sponge *Hymedesmia coriacea* sampled after 4 days exposure to ^13^C and ^15^N labeled cold-water coral mucus. Data presented as mean ± SD*. *Note that the cold-water sponge tissue data are from three sponge specimens maintained in a single experimental chamber during the labeling procedure. Subsequent detritus production incubations were conducted in individual chambers.

After the stable isotope labeling procedure the WW and CW corals produced and released mucus that was enriched in both ^13^C and ^15^N. WW coral mucus was enriched by 913 ± 250% for δ^13^C and 3518 ± 1360% for δ^15^N, while the CW mucus was enriched by 492 ± 212% for δ^13^C and 3219 ± 536% for δ^15^N (Fig. 1; data presented as mean ± SD). The C:N ratio of the WW mucus (12.7 ± 1.1) was twice as high as the CW mucus (6.1 ± 0.4; GLM: *F*_1,19_ = 196.5, *p* < 0.001). Overall, the total labeling level of the WW coral mucus was 1.0 atm% ^13^C and 1.4 atm% ^15^N and the CW coral mucus was labeled with 0.4 atm% ^13^C and 1.0 atm% ^15^N. This was sufficient to trace subsequent δ^13^C and δ^15^N enrichment in the sponge tissue and sponge detritus (Fig. 1), enabling calculation of the total mucus-derived C (^13^C + ^12^C) and N (^15^N + ^14^N) incorporated into the sponge tissue and sponge detritus.

### Incorporation of coral mucus C and N into sponge tissue

**Figure 2:**
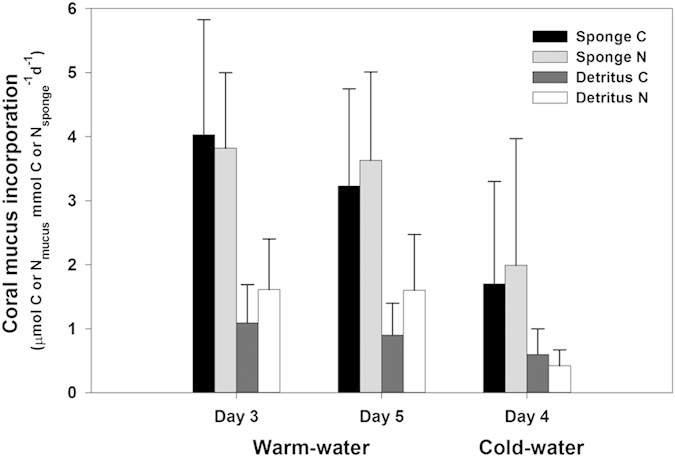
Processing of coral mucus C and N by the warm-water sponge *Mycale fistulifera* and the cold-water sponge *Hymedesmia coriacea*. Data presented as daily incorporation rates (mean ± SD) of coral mucus C and N assimilated into sponge tissue (μmol C or N_mucus_ mmol C or N_sponge_^−1^ d^−1^), and daily release rates of coral mucus C and N in sponge detritus (μmol C or N_mucus_ mmol C or N_sponge_^−1^ d^−1^). Rates shown for *M. fistulifera* after 3 and 5 days exposure to labeled warm-water coral mucus and for *H. coriacea* after 4 days exposure to labeled cold-water coral mucus*. *Note that the cold-water sponge tissue data are from three sponge specimens maintained in a single experimental chamber during the labeling procedure. Subsequent detritus production incubations were conducted in individual chambers.

Both the WW sponge *M. fistulifera* and CW sponge *H. coriacea* displayed enrichment of δ^13^C and δ^15^N in their tissues, indicating uptake of the coral mucus (Fig. 1). On average, *M. fistulifera* assimilated 3.6 ± 1.7 μmol C_mucus_ mmol C_sponge_^−1^ d^−1^ and 3.7 ± 1.2 μmol N_mucus_ mmol N_sponge_^−1^ d^−1^. The CW sponge *H. coriacea*, incorporated coral mucus at lower but comparable rates of 1.7 ± 1.6 μmol C_mucus_ mmol C_sponge_^−1^ d^−1^ and 2.0 ± 2.0 μmol N_mucus_ mmol N_sponge_^−1^ d^−1^, although it should be noted these rates are based on three specimens from a single experimental chamber (Fig. 2). The WW sponge *M. fistulifera*, exhibited an increase in δ^13^C and δ^15^N values compared to background values (i.e. the Δδ^13^C and Δδ^15^N) from day 3 to day 5, indicating the accumulation of mucus-derived C and N over time (Fig. 1). However, this increase was significant only for mucus N (*F*_1,16_ = 7.3, *p* = 0.02). Additionally, there was no significant difference in the actual incorporation rate of mucus-derived C and N by *M. fistulifera* on day 3 compared to day 5 (*F*_1,16_ = 1.6, *p* = 0.32 and *F*_1,16_ = 0.001, *p* = 0.98 for C and N, respectively) indicating that the sponge accumulated mucus C and N at a constant daily rate (Fig. 2). There were also no significant differences between the three WW aquarium set-ups (*F*_1,16_ = 2.3, *p* = 0.15 and *F*_1,16_ = 1.5, *p* = 0.24 for C and N, respectively), demonstrating comparable conditions in all tanks (i.e. no “tank” effect) and that sponge uptake rates were similar despite any potential differences in the amount of coral mucus supplied between tanks.

**Figure 3:**
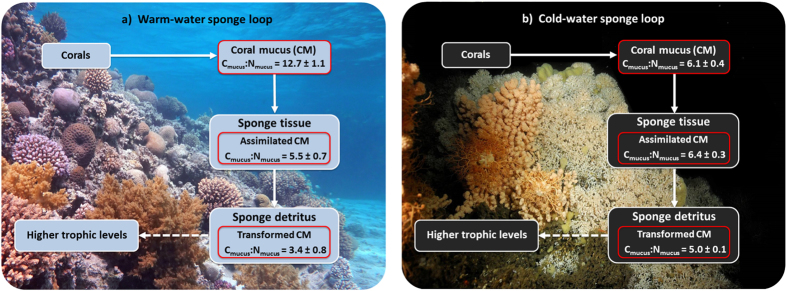
Transfer of coral mucus C and N in a) the warm-water sponge loop and b) the cold-water sponge loop. C:N ratios presented inside red boxes (i.e. C_mucus_:N_mucus_) indicate the C:N ratio of coral mucus-derived organic matter that is transferred at each step of the sponge loop. Solid lines indicate trophic transfer of coral mucus confirmed in the current study and dotted lines indicate trophic transfers inferred from literature. Note that C_mucus_:N_mucus_ refers only to the ratio of mucus-derived C and N transferred at each step and not to the C:N ratio of the bulk sponge tissue and detritus. Photograph in (a) © Malik Naumann and (b) © Solvin Zankl.

Despite being supplied with coral mucus with a high initial C:N ratio of 12.7 ± 1.1, the C:N ratio of mucus-derived organic matter incorporated into the tissue of the WW sponge *M. fistulifera* (i.e. C_mucus_:N_mucus_) was lower at 5.5 ± 0.7 (Fig. 3). However, it should be noted that respiration of mucus C was not quantified here. By contrast, the CW sponge *H. coriacea* assimilated mucus-derived organic matter at a higher C:N ratio of 6.4 ± 0.3 (Fig. 3), similar to the C:N ratio of the coral mucus supplied (6.1 ± 0.4).

### Transfer of coral mucus C and N into sponge detritus

The transfer of coral mucus C and N through sponges into sponge detritus is shown by the production of ^13^C- and ^15^N-enriched detritus after exposure to labeled coral mucus (Fig. 1). The WW sponge, *M. fistulifera* converted coral mucus C and N into detritus at rates of 0.9 ± 0.5 μmol C_mucus_ mmol C_sponge_^−1^ d^−1^ and 1.5 ± 0.8 μmol N_mucus_ mmol N_sponge_^−1^ d^−1^, with no significant differences between day 3 and day 5 and no significant tank effect (Fig. 2). The CW sponge *H. coriacea*, transformed coral mucus C and N into detritus at rates of 0.6 ± 0.4 μmol C_mucus_ mmol C_sponge_^−1^ d^−1^ and 0.4 ± 0.3 μmol N_mucus_ mmol N_sponge_^−1^ d^−1^ (Fig. 2). While *M. fistulifera* released mucus-derived N at a significantly higher rate than C (*F*_1,16_ = 13.3, *p* = 0.002), there was no difference in the release of mucus-derived C and N by *H. coriacea*.

When tracing the coral mucus C and N through the sponge tissue into the sponge detritus, both sponges produced detritus with a lower C:N ratio of mucus-derived organic matter (i.e. C_mucus_:N_mucus_) compared to the sponge tissue (Fig. 3). Similar to the tissue results however, the C:N ratio of mucus-derived organic matter in the detritus of *M. fistulifera* (3.4 ± 0.8) was lower compared to *H. coriacea* (5.0 ± 0.1; Fig. 3). Thus, the WW sponge *M. fistulifera* preferentially incorporated and transferred coral mucus N, decreasing the C:N ratio of mucus-derived organic matter at each step of the sponge loop (Fig. 3).

Overall, the WW sponge *M. fistulifera* converted 21 ± 11% of the assimilated mucus C and 32 ± 10% of the assimilated mucus N into detritus C and N, releasing a significantly higher percentage of mucus N (*F*_1,33_ = 4.548, *p* = 0.04). Compared with the WW sponge, the CW sponge *H. coriacea* transformed a similar percentage of assimilated mucus N (39 ± 10%) into detritus, but a higher amount of mucus C (40 ± 29%). However, the standard deviation of the CW data was high and more replication is needed to confirm mucus-to-detritus C conversion rates in CW sponges.

### Sponge versus associated bacterial specific incorporation of coral mucus C

**Figure 4:**
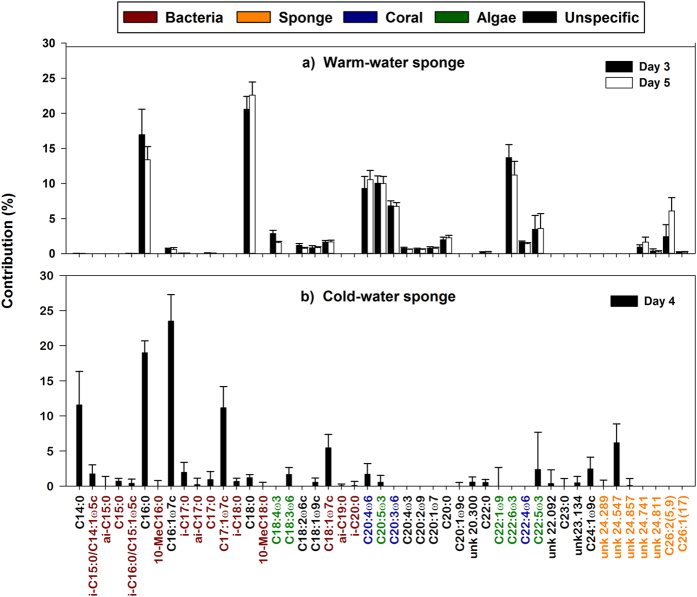
Distribution of coral mucus C in sponge phospholipid fatty acids (PLFAs). Data presented as % of total coral mucus C assimilated into PLFAs (mean ± SD) in a) the warm-water sponge *Mycale fistulifera,* after 3 and 5 days exposure to labeled warm-water coral mucus, and b) the cold-water sponge *Hymedesmia coriacea,* after 4 days exposure to labeled cold-water coral mucus*. *Note that the cold-water PLFA data are from three sponge specimens maintained in a single experimental chamber during the labeling procedure.

The coral mucus C assimilated by the WW and CW sponges was partly incorporated into phospholipid-derived fatty acids (PLFAs), demonstrating active processing of coral mucus (Fig. 4). The WW sponge *M. fistulifera* incorporated 1.8 ± 0.3% of the assimilated mucus C into PLFAs, with no significant difference in the daily PLFA incorporation rates on day 3 and day 5 (0.07 ± 0.03 μmol C_mucus_ mmol C_sponge_^−1^ d^−1^ and 0.06 ± 0.02 μmol C_mucus_ mmol C_sponge_^−1^ d^−1^, respectively) and no significant tank effect. The CW sponge *H. coriacea* transferred a similar percentage (1.9 ± 0.4%) of the total assimilated mucus C into PLFAs at a comparable rate of 0.05 ± 0.03 μmol C_mucus_ mmol C_sponge_^−1^ d^−1^.

**Figure 5:**
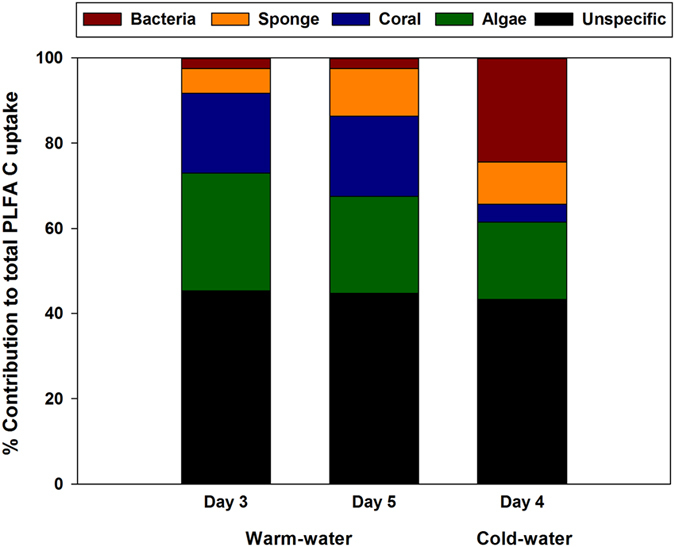
Percent distribution of coral mucus C assimilation into bacterial, sponge, coral, and algal phospholipid fatty acids (PLFAs). Data shown for warm-water sponge *M. fistulifera* after 3 and 5 days exposure to labeled coral mucus, and for the cold-water sponge *H. coriacea* after 4 days exposure to labeled coral mucus*. *Note that the cold-water PLFA data are from three sponge specimens maintained in a single experimental chamber during the labeling procedure.

In both species, mucus-derived C could be traced into PLFAs identified as bacterial, coral, algal or sponge biomarkers (Fig. 4). In addition to known sponge PLFAs, such as C26:2(5,9), both species contained a number of unidentified long chain (>C:24) PLFAs characteristic of demosponges^38^, that were therefore considered sponge biomarkers (Fig. 4). In the WW sponge *M. fistulifera,* the amount of mucus-derived C traced into these sponge biomarkers increased from 6% on day 3 to 11% on day 5, although this increase was not significant (Fig. 5). A similar percentage (10%) was traced into sponge biomarkers in the CW sponge *H. coriacea* (Fig. 5). Typical bacterial PLFAs, including iso-, anteiso-, methyl-branched, and odd numbered branching PLFAs^39^ (Fig. 4), accounted for only 2% of the total coral mucus C assimilated into PLFAs by *M. fistulifera*, but 24% for *H. coriacea* (Fig. 5), suggesting higher uptake of coral mucus by sponge-associated bacteria in the CW sponge. Additionally, mucus C was traced into PLFAs likely originating from the mucus-producing coral hosts, including typical coral biomarkers C20:3ω6, C20:4ω6 and C22:4ω6^7^,^40^,^41^, but also common algal biomarkers C18:4ω3, C20:5ω3, and C22:6ω3 (Fig. 4). For the WW coral, these algal biomarkers originate from their symbiotic zooxanthellae^40^,^41^, as the zooxanthellae were responsible for the photosynthetic uptake of ^13^C -NaHCO_3_ and ^15^N -NaNO_3_, which are then transferred to the coral host. The heterotrophic CW corals were initially fed with ^13^C- and ^15^N-labeled diatoms, explaining the presence of algal biomarkers. PLFAs of coral-host origin accounted for 42–46% of the coral-derived C traced into the PLFA fraction in the WW sponge, *M. fistulifera*, higher than the 22% for the CW sponge *H. coriacea* (Fig. 5). In *M. fistulifera*, typical coral PLFAs also accounted for 10–17% of the total PLFAs in the unlabeled control sponges, suggesting that coral mucus could be a source of dietary PLFAs in *M. fistulifera*. In *H. coriacea*, the majority (80 ± 12%) of the PLFAs in the control sponges were identified as sponge biomarkers resulting from *de novo* synthesis and modification of dietary PLFAs by the sponge. However, coral biomarkers were also present and accounted for 4 ± 3% of the total PLFA content.

## Discussion

By demonstrating the uptake of coral mucus by warm- and cold-water reef sponges and its subsequent transformation into sponge detritus, we provide the first evidence that the sponge loop recently identified in the Caribbean^27^, functions not only within shallow-water reef ecosystems of other oceanic regions (i.e. Red Sea, Indo-Pacific), but even in cold-water reefs of the deep sea. Despite pronounced differences in the environmental characteristics of warm-water (WW) and cold-water (CW) reef ecosystems, both sponges assimilated and transformed coral mucus C and N into particulate detritus at remarkably similar rates. Previously, the sponge loop has only been demonstrated using laboratory-produced diatom DOM. Therefore, we not only extend the spatial validity of the sponge loop, but also demonstrate its functioning using a resource naturally produced on the reef: coral mucus. Importantly, this elucidates a direct trophic link between two fundamental reef ecosystem engineers, scleractinian corals and sponges, on both shallow WW and deep-sea CW reefs and identifies a new trophic pathway for the transfer of coral-derived organic matter in reef ecosystems, providing an additional mucus-recycling pathway to the established microbial loop^18^,^25^.

The similar rate of assimilation of coral mucus into the bulk tissue of the two sponges indicates that WW and CW sponges may have a comparable capacity for mucus uptake. Based on natural abundance stable isotope signatures, coral mucus has been inferred to be a major component (48–73%) of the diet of Caribbean cavity sponges^42^, but our findings provide unequivocal and quantitative evidence for the uptake and assimilation of coral mucus by sponges originating from shallow-water and deep-sea reef habitats. Furthermore, the assimilation of coral mucus C into sponge phospholipid-derived fatty acids (PLFAs) synthesized *de novo* or by modification of coral-derived PLFAs demonstrates that sponges actively process coral mucus similarly to algal and bacterial food sources, confirming its nutritional value^37^,^43^. Given the dominant coral cover and high release rates of coral mucus on Tisler Reef (Norwegian Shelf)^18^ and in the Red Sea^20^, as well as the close proximity of the two sponge species to corals in their respective habitats^6^,^44^, coral mucus likely represents a readily available food source for the two investigated sponges. Considering the extremely oligotrophic conditions in the Red Sea^44^,^45^ and the spatially and temporally variable input of organic matter to CW reefs^12^,^14^,^46^, the ability to utilize coral mucus as a reef-produced resource may be an advantageous strategy for reef sponges. Moreover, this trophic link provides a key ecosystem function by retaining coral mucus, an energy-rich resource^1^,^16^, within the reef ecosystem, thereby preventing the loss of the energy and nutrients bound in coral mucus to the adjacent ocean. In combination with microbial degradation processes, uptake by sponges may ensure high retention of coral mucus C and N within WW and CW reef ecosystems. Therefore, the importance of coral mucus to the overall sponge diet and the magnitude of coral mucus uptake by the sponge community at the ecosystem scale should be determined.

Both the WW and CW sponge release substantial fractions of assimilated coral mucus C and N as sponge detritus: 21% C and 32% N for *M. fistulifera* and 40% C and 39% N for *H. coriacea*. These rates are comparable to detritus conversion rates of Caribbean sponges that released 11–24% (C) and 18–36% (N) of assimilated diatom DOM^27^, demonstrating that release as detritus is the fate of a substantial fraction of organic matter assimilated by sponges in both WW and CW reef ecosystems and may represent a significant flow of energy and nutrients within coral reef trophic webs. Recycling via the sponge loop is estimated to approach reef gross primary production, exceeding recycling by the microbial loop by an order of magnitude^27^. Mucus release rates by WW and CW corals are comparable^17^,^20^,^21^ and sponges are also highly abundant on CW reefs^47^,^48^,^49^. Given the similar coral mucus uptake and transformation rates by the two sponges, the sponge loop may be quantitatively important for the recycling of energy and nutrients on CW reefs. However, due to differences in the ambient availability of C and N on WW and CW reefs (Supplementary Table S1), we hypothesize that the functional role of the sponge loop differs between the two ecosystems. Due to the low N availability on oligotrophic WW reefs such as in the Red Sea^45^, the retention and recycling of N may be particularly important for supporting primary productivity and ecosystem function. By contrast, on CW reefs, organic C input arrives in pulses creating periods of high food availability intermitted by periods of low organic C availability resulting in C (energy) limitation^14^,^50^. While the mechanisms behind this benthic-pelagic coupling have been intensively studied^12^,^13^,^14^, little is known how recycling within the reef community may help bridge periods of low food supply. By retaining coral mucus and transferring its energy and nutrient content into the reef food web, the enhancement of secondary production by the sponge loop may contribute to sustaining the CW reef community during periods of low food supply. Therefore, we suggest that N retention in WW and C retention in CW reefs may disproportionately contribute to ecosystem functioning. This is tentatively supported by the relatively higher uptake and transfer of N at each step of the WW sponge compared to the CW sponge loop, as evidenced by the substantial decrease in the C:N ratio of mucus-derived organic matter (i.e. C_mucus_:N_mucus_) within the WW sponge loop (Fig. 3). However, due to the low sample size of the CW data, as well as the lack of information on the composition of the released WW and CW coral mucus, further investigation is required to quantify the functional importance of the sponge loop in contributing to the efficient biogeochemical cycling typical of WW and CW coral reef ecosystems.

Sponges host diverse microbial populations and can be classified into high microbial abundance (HMA) and low microbial abundance (LMA) species based on the number of associated microbes in their tissue^51^. Since bacteria are considered the main consumers of DOM in the ocean, sponge-associated bacteria are suspected to play an important role in DOM uptake by sponges, with higher uptake predicted for HMA compared to LMA sponges^34^,^52^. Higher incorporation into bacterial PLFAs in *H. coriacea* (24%) compared to *M. fistulifera* (2%) suggests bacteria were more active in coral mucus uptake in the CW sponge. However, both sponge species belong to the order Poecilosclerida, which appears to exclusively consist of LMA species^51^. There was also no significant difference in the overall coral mucus incorporation between the two sponges, and mucus-derived C was also incorporated into sponge-specific PLFAs (5–10%). This suggests the involvement of sponge cells in coral mucus uptake, particularly in *M. fistulifera* where associated bacteria appeared to play a minor role. Moreover, high DOM uptake, also into sponge-specific PLFAs, is known for several LMA species^27^,^31^,^33^,^37^ reviewed by Pawlik *et al.*^53^, indicating DOM uptake is not limited to HMA sponges.

Detritus production by sponges is a phenomenon that has been observed in tropical^27^,^36^ and deep-sea sponges^54^; but the mechanisms by which sponges produce detritus are not fully understood. Rapid cell-turnover and subsequent shedding is believed to be a key source of sponge detritus, although excretory by-products also contribute^35^,^36^,^55^. Interestingly, we found that both species produced detritus that was relatively enriched in coral mucus-derived N (i.e. lower C_mucus_:N_mucus_ ratio) compared to the sponge tissue (Fig. 3), a phenomenon also observed for Caribbean sponges fed with diatom DOM^27^. Consequently, sponges not only transform mucus C and N into detrital C and N—enabling its transfer to reef fauna otherwise unable to directly utilize it—but they also modify the relative availability of this C and N for higher trophic levels and may, therefore, provide a high quality food source. Since corals release a large fraction of their assimilated C as mucus^22^,^23^, this may be a key mechanism by which the substantial energy and nutrients harvested by corals can be transferred to other reef fauna. On Caribbean reefs, sponge detritus is rapidly utilized by a variety of benthic detritivores^27^. On CW coral reefs, the consumption of sponge detritus has not been established, but detritivores are an important component of CW benthic communities^48^,^50^. Furthermore, trophic models indicate that detritus can account for 51% of the total C ingested by CW reef communities^56^, suggesting sponge detritus may be widely utilized by CW reef fauna.

While the transfer of coral mucus C and N to higher trophic levels via sponge detritus remains to be confirmed, our findings strongly suggest coral mucus fuels a sponge loop in both WW and CW reef ecosystems. This trophic pathway has the potential to substantially impact our understanding of food web dynamics and biogeochemical cycles on coral reefs. A recent study suggests that phase-shifts from coral to macroalgal dominance on degraded coral reefs could impact fish productivity on WW reefs by altering DOM availability for the sponge loop^57^, demonstrating the potential for the sponge loop to impact the entire coral reef food web. Additional environmental and human impacts, such as increased sedimentation and ocean acidification may lead to changes in mucus production^58^,^59^, and could thereby further influence organic matter transformation by the sponge loop. Given the potential importance of the sponge loop to ecosystem function on WW and CW reefs, future studies should quantify the magnitude of recycling by the sponge loop to evaluate its role in trophodynamics and biogeochemical cycling in these complex reef ecosystems.

## Materials and Methods

### Sample collection and maintenance

WW corals and sponges were collected from the Marine Science Station (MSS) reef, Aqaba, Jordan (29°27’ N, 34°58’ E) located in the northern Gulf of Aqaba, Red Sea. Free-living corals from the genera *Fungia, Ctenactis,* and *Herpolitha* (Scleractinia, Fungiidae) were collected from the reef between 8–20 m water depth by SCUBA. These genera can be removed from the reef without causing physical damage, are locally abundant, and produce large quantities of mucus^60^. The encrusting sponge *Mycale fistulifera* was selected as a model species since it was the most abundant sponge species at 10 m water depth at the study site (accounting for 65% of the non-cryptic sponge cover)^44^, and is found in close proximity to living corals. Fragments of *M. fistulifera* were collected between 8–12 m water depth by chiselling fragments of dead coral skeleton overgrown by the sponge. Corals and sponges were immediately transferred to the aquarium facilities at the MSS without air exposure. Sponge specimens were trimmed to approximately the same size (0.08 ± 0.04 g DW sponge^−1^), cleaned of epibionts, and attached to ceramic tiles with coral glue. Corals and sponges were maintained in 100-L flow-through aquaria supplied with seawater pumped directly from the reef at 10 m water depth at a flow rate of ~10 L min^−1^. Natural light levels were adjusted to *in situ* levels at ~15 m water depth (~120 μmol photons m^−2^ s^−1^ PAR) using layers of black mesh and parallel *in situ* and aquarium measurements of photosynthetically active radiation (PAR μmol photons m^−2^ s^−1^, wavelength 400–700 nm) using a quantum sensor (Model LI-192SA; Li-Cor). Sponges were acclimated for 1 week prior to the start of experiments and only healthy individuals that were actively pumping were used. Corals were acclimated for at least 72 h.

The CW coral *Lophelia pertusa* and encrusting sponge *Hymedesmia coriacea* were collected from Tisler Reef, located at 70–155 m water depth in the Skagerrak at the border between Norway and Sweden (58°59’ N 10°58’ E). *L. pertusa* is the dominant reef-building coral on Tisler Reef, while sponges of the genus *Hymedesmia* are widespread in CW reef ecosystems with *H. coriacea* a locally abundant species commonly found in close contact with *L. pertusa*^6^,^49^. Specimens were collected from distinct coral and sponge individuals at water depth of 110 m using the remotely operated vehicle Sperre Subfighter 7500 DC and transported in cooling boxes filled with cold seawater (7–8 °C) within a few hours to the laboratory facilities at the Sven Lovén Centre in Tjärnö, Sweden. Sponge specimens were trimmed to approximately the same size (0.04 ± 0.02 g DW sponge^−1^) and cleared of epibionts. All specimens were maintained in flow-through aquaria (~20 L) with sand-filtered water pumped from 45 m depth from the Koster-fjord at a rate of ~1 L min^−1^. Aquaria were kept in a dark climate-controlled room at 7 °C corresponding to *in situ* temperatures on Tisler reef (6–9 °C). Coral specimens were acclimated for 6 weeks and sponge specimens for 1 week prior to experimentation in order to allow full recovery, and only healthy specimens were used.

### Experimental design

A flow-chart (Fig. S1) provides an overview of all consecutive experimental steps and work phases, which are described in detail below.

### Coral labeling

WW corals were enriched with ^13^C and ^15^N by addition of ^13^C-NaHCO_3_ and ^15^N-NaNO_3_ label compounds (Cambridge Isotope Laboratories, 99% ^13^C and 98% ^15^N), which are taken up and transferred to the coral host via its photosynthetic endosymbionts (i.e. zooxanthellae)^60^. For 8 days, each morning at 08:00 the inflows to the coral aquaria were stopped and 36 mg L^−1^ NaH^13^CO_3_ and 1 mg L^−1^ Na^15^NO_3_ were added to each aquaria. Aquaria pumps maintained water circulation and air exchange for the 8-h labeling period and the flow-through was resumed over-night. A flow-through water bath maintained temperatures in the aquaria within ± 1 °C of the ambient flow-through water temperature (26.7–27.5 °C).

CW corals were enriched with ^13^C and ^15^N by repeated feeding with ^13^C and ^15^N-enriched diatoms, a food source readily assimilated by the *L. pertusa*^7^. Isotopically-labeled diatoms were produced by injecting a sterile inoculum of the diatom *Thalassiosira pseudonana* into an f/2 culture medium composed of 80% ^13^C -NaHCO_3_ and 70% ^15^N -NaNO_3_ (Cambridge Isotope Laboratories, 99% ^13^C, 99% ^15^N). The diatoms were axenically (i.e. bacteria-free) cultured for 3 weeks at a 12-h light-dark cycle and then concentrated by centrifugation at 450 *g*, rinsed three times with 0.2-μm filtered seawater to remove residual label, and stored frozen until use. Corals were incubated in 10-L incubations chambers and fed the enriched diatoms at a concentration of 1.6 mg C L^−1^ d^−1^ and 0.3 mg N L^−1^ d^−1^ for 3 weeks. Water in the incubation chambers was exchanged every 12 h to prevent accumulation of waste products.

### Transfer of coral mucus to sponges

For the WW experiment, the transfer of coral mucus to *M. fistulifera* was investigated using six two-tiered flow-through aquaria set-ups, each consisting of a paired upper and lower aquarium connected via flow-through. The six upper aquaria (100 L) were supplied with fresh flowing seawater pumped directly from the reef (10 m water depth) at a flow rate of ~10 L min^−1^. Within these six aquaria, isotopically-labeled fungiid corals (ten individuals per aquaria) were maintained in three of the upper aquaria (*n* = 3) while the additional three upper aquaria without labeled corals served as controls (*n* = 3). Water from each upper aquaria flowed into the corresponding lower aquaria (100 L) below. The lower aquaria each contained six sponge specimens so that the treatment sponges were continuously supplied with water exposed to the labeled corals. Artificial aquaria lights provided the corals with ~120 μmol quanta m^−2^ s^−1^ PAR and aquaria circulation pumps (~150 L h^−1^) enhanced water-flow. To investigate the incorporation of mucus into sponge tissue over time, three of the six sponge specimens from each tank were collected after three days exposure to the labeled corals and the remaining sponges were collected after five days exposure (*n* = 3 tank replicates each with three sponge specimens per time point). The collected sponges were removed from the labeling set-up and rinsed in label-free flowing seawater for 10 min. Each sponge specimen was then transferred to an individual 2 L incubation chamber filled with fresh label-free seawater, and incubated for 3 h to determine the production of sponge detritus. At the end of the incubation, the sponges were removed and the incubation water (~1.8 L) was filtered onto pre-combusted (450 °C, 4 h) GF/F filters to collect the produced particulate organic matter (POM). Filters were then dried at 40 °C for 48 h. Sponge tissue was removed from the attached substrate with a sterile scalpel blade and stored frozen in pre-combusted glass vials at −80 °C until further processing. On days one and five, three corals per tank were removed from the experimental set-up, rinsed in label-free seawater and air exposed for mucus production (2 min). The collected mucus was frozen at −80 °C for δ^13^C and δ^15^N determination.

For the CW experiment, the transfer of coral mucus to *H. coriacea* was investigated using one set-up of paired cylindrical incubation chambers filled with GF/F filtered water pumped from 45 m in the Koster-fjord. Isotopically-labeled *L. pertusa* fragments (75 g DW coral total) were placed in the first chamber (10 L), while the second chamber (10 L) contained three sponges (*n* = 1 chamber replicate with three sponge specimens). It should be noted that the use of a single experimental paired-chamber set-up means that the CW sponge replicates are pseudo-replicates, and therefore no statistical comparisons were performed on the sponge uptake data. The coral chamber was connected to the sponge chamber by a set of two tubes and water was re-circulated between the two chambers at a rate of 200 mL min^−1^ via a pump system. The coral chamber was equipped with an additional pump (150 L h^−1^) to enhance water circulation. Every 24 h, half the water in the set-up (5 L) was replaced with fresh seawater to prevent the accumulation of metabolic waste products. Three control sponge specimens were incubated in parallel without labeled corals. At the beginning and end of the experiment, water samples were taken from the coral chamber and filtered onto pre-combusted GF/F filters for mucus δ^13^C and δ^15^N determination. After 4 days, each sponge fragment was transferred to an individual 1-L incubation chamber containing fresh-filtered (GF/F) label-free seawater and incubated for 24 h to determine the production of sponge detritus. At the end of the incubation the sponge fragments were removed and the water was filtered onto pre-combusted GF/F filters to collect the produced POM. All POM filters, coral tissue, and sponge tissue samples were frozen at −20 °C until further processing.

### Sample treatment and analysis

WW and CW sponge tissues were lyophilized. Dried sponge tissue samples were weighed and homogenized by mortar and pestle. Subsamples of sponge tissue and dried POM filters were weighed and transferred to silver boats for bulk δ^13^C and δ^15^N isotope analysis. All samples for δ^13^C were decalcified by acidification with HCl for analysis of the organic C content. Isotopic ratios and total C_organic_ and N content were measured simultaneously using a THERMO NA 2500 elemental analyzer (EA) coupled to a THERMO/Finnigan MAT Delta plus isotope ratio mass spectrometer (IRMS) via a THERMO/Finnigan Conflo III- interface (WW) and a Thermo Electron Flash 1112 EA coupled to a Delta V IRMS (CW).

Carbon and nitrogen stable isotope ratios are expressed in delta notation as: δ^13^C or δ^15^N (%) = (R_sample_/ R_ref_  − 1) × 1000, where R_sample_ is the ratio of heavy/light isotope (^13^C/^12^C or ^15^N/^14^N) in the sample and R_ref_ is the heavy/light isotope ratio of the reference material, the Vienna Pee Dee Belemnite standard for C (R_ref_ = 0.01118) and atmospheric nitrogen for N (R_ref_ = 0.00368 N). The incorporation of excess (i.e. above background) ^13^C and ^15^N is expressed as the specific enrichment, i.e. Δδ^13^C and Δδ^15^N values (Fig. 1). This was calculated by subtracting the background δ^13^C and δ^15^N values of the control sponges from the values of the sponges exposed to labeled corals. The fractional abundance (F) of the heavy isotope in the sample (^13^C/[^13^C + ^12^C] or ^15^N/[^15^N + ^14^N]) was calculated as F_sample_ = R_sample_/(R_sample_ + 1) and reported as atom percent (atm % = F × 100). The excess fractional abundance of heavy isotope (E) was calculated as the difference between the F_sample_ and F_background_: E = F_sample_ – F_background_. Incorporation rates are the total uptake (I), calculated by multiplying E by the C_org_ or N content of the sponge tissue or detritus (Fig. 2) or PLFA-C content (Fig. 4). To correct for the different enrichment of the WW and CW coral mucus and to determine the total mucus-derived C (^12^C + ^13^C) and N (^14^N + ^15^N) incorporated, the total uptake (I) was divided by the fractional abundance of the coral mucus supplied to the sponges. Rates were then normalized to time and the C_org_ or N content of the sponges and reported as μmol C_mucus_ mmol C_sponge_^−1^ d^−1^and μmol N_mucus_ mmol N_sponge_^−1^ d^−1^ (mean ± SD) as per de Goeij *et al.*^27^. To determine the C:N ratios of coral mucus-derived organic matter incorporated into the sponge tissue and detritus (i.e. C_mucus_:N_mucus_; Fig. 3), the total amount of coral mucus-derived C incorporated was divided by the total amount of coral mucus-derived N incorporated.

### Phospholipid fatty acid analysis

Phospholipid-derived fatty acids (PLFAs) of the sponge samples (~0.018 g) were extracted according to Boschker *et al.*^61^. Total fatty acids were extracted using a modification of the Bligh & Dyer method^62^ and separated on a silicic-acid column (Merck Kieselgel 60) to obtain the PLFAs, which were then derivatized by mild alkaline transmethylation to generate fatty acid methyl esters (FAMEs). Concentration and C isotopic composition of individual FAMEs were determined with a gas-chromatograph combustion interface isotope ratio mass spectrometer (GC-c-IRMS). Identification of individual FAMEs was based on the comparison of retention times with known standards using columns with different polarity and use of GC-MS, if needed.

### Data analysis

For the WW data (*n* = 3 tank replicates each with three sponge specimens), statistical differences were tested using General Linear Models (GLMs). First a GLM with “tank” as the fixed factor was performed to test for potential tank effects. Since “tank” was not found to be a significant factor, it was excluded from subsequent models and each sponge specimen was considered as an individual replicate (*n* = 9). To confirm assumptions of normally distributed and homogenous residuals, qqplots and scatter plots of residuals against fitted values were visually inspected, and data were log-transformed where necessary. Due to the low sample size of the CW data (*n* = 1 chamber replicates with three sponge specimens), which can only be considered as pseudo-replicates, no statistical comparisons were made for the CW data or between the WW and CW data. All statistical tests were carried out in R v. 3.1.1 (R Development Core Team, 2014).

## Additional Information

**How to cite this article**: Rix, L. *et al.* Coral mucus fuels the sponge loop in warm- and cold-water coral reef ecosystems. *Sci. Rep.* **6**, 18715; doi: 10.1038/srep18715 (2016).

## Supplementary Material

Supplementary Information
