# Supplementary material for: Coral mucus fuels the sponge loop in warm- and cold-water coral reef ecosystems
Source: Sci Rep. 2016 Jan 7;6:18715. doi: 10.1038/srep18715 (PMC4703987; doi:10.1038/srep18715)
Supplement: Supplementary Information [file srep18715-s1.pdf]

# Coral mucus fuels the sponge loop in warm- and cold-water coral reef ecosystems

Laura Rix[1*], Malik S. Naumann[1], Jasper M. de Goeij[2], Christina E. Mueller[3], Ulrich Struck[4], Jack J. Middelburg[5], Fleur C. van Duyl[6], Fuad A. Al-Horani[7], Christian Wild[1,8], Dick van Oevelen[3]

[1]Coral Reef Ecology Group (CORE), Leibniz Center for Tropical Marine Ecology (ZMT), Fahrenheitstr. 6, 28359 Bremen, Germany

[2]Department of Aquatic Environmental Ecology, Institute for Biodiversity and Ecosystem Dynamics, University of Amsterdam, PO Box 94248, 1090 GE Amsterdam, the Netherlands

[3]Royal Netherlands Institute for Sea Research (NIOZ-Yerseke), PO Box 140, 4400 AC Yerseke, the Netherlands

[4]Museum für Naturkunde, Leibniz Institute for Evolution and Biodiversity Science, Invalidenstr. 43, 10115 Berlin, Germany

[5]Department of Earth Sciences – Geochemistry, Utrecht University, PO Box 80.021, 3508 TA Utrecht, the Netherlands

[6]Royal Netherlands Institute for Sea Research (NIOZ-Texel), PO Box 59, 1790AB Den Burg, Texel, the Netherlands

[7]The University of Jordan – Aqaba and Marine Science Station (MSS), PO Box 2595, Aqaba 77110, Jordan

[8]Faculty of Biology and Chemistry (FB 2), University of Bremen, NW 2 / Leobener Str., 28359 Bremen, Germany

*corresponding author, E-mail: laura.n.rix@gmail.com

# Supplementary Information:

## Supplementary Figures:

**Supplementary Figure S1: Flow-chart describing the three phases of the warm-water (WW) and cold-water (CW) stable isotope-tracer experiments.** Phase 1 describes the labeling of the WW and CW corals with $^{13}C$ and $^{15}N$ tracers, Phase 2 outlines the transfer of coral mucus-derived C and N from the $^{13}C$ and $^{15}N$-labeled corals into the sponge tissues in aquaria flow-through set-ups (WW: *n* = 3 aquaria replicates each with three sponge specimens per treatment) or a recirculation chamber set-up (CW: *n* = 1 chamber set-ups with three sponge specimens per treatment), and Phase 3 shows the transfer of coral mucus-derived C and N from the $^{13}C$ and $^{15}N$-labeled sponges to the sponge detritus in individual incubation chambers (WW: *n* = 9, CW: *n* = 3).

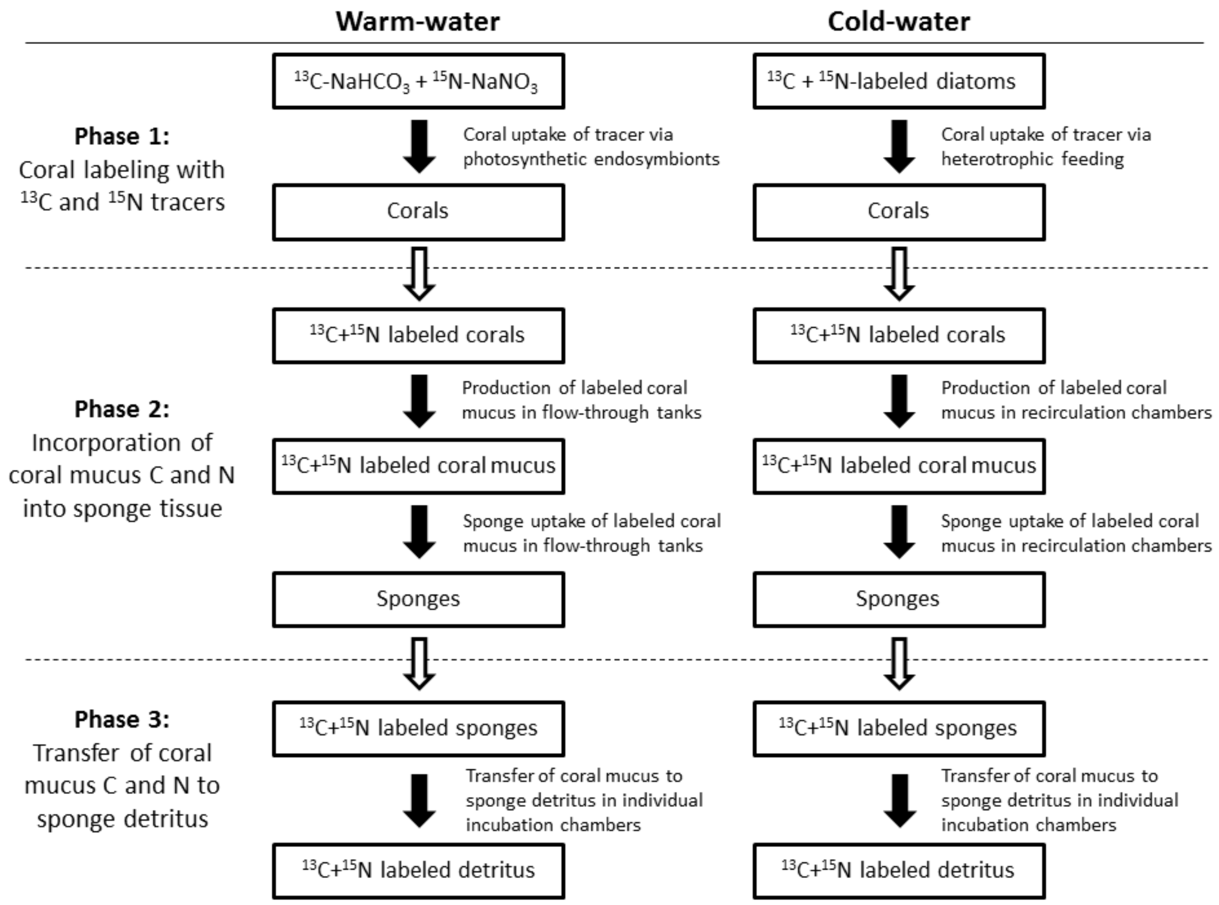

# Supplementary Tables

**Supplementary Table S1. Environmental parameters characteristic of warm-water (WW), Red Sea coral reefs and cold-water (CW), north Atlantic *Lophelia pertusa* reefs.** Parameters include dissolved inorganic nitrogen (DIN), soluble reactive phosphorus (SRP), dissolved organic carbon (DOC), particulate organic carbon (POC), particulate nitrogen (PN), and chlorophyll a (Chl *a*). [a] indicates the inorganic nutrient supply limiting WW coral growth and [b] the organic nutrient supply limiting CW coral growth.

| Parameter | Warm-water Red Sea coral reefs | Cold-water North Atlantic coral reefs |
|---|---|---|
| Depth (m) | 1 – > 100[1] | 50 – > 1000[2] |
| Temperature (°C) | 21 – 29[3] | 6 – 10[2,4,5] |
| DIN (μmol $L^{-1}$)[a] | 0.2 – 1.1[6] | 2.2 – 19.1[2] |
| SRP (μmol $L^{-1}$)[a] | 0.04 – 0.1[6] | 0.3 – 3.6[2] |
| DOC (μmol $L^{-1}$) | 76 – 87[7] | 51 – 73[8] |
| POC (μmol $L^{-1}$)[b] | 6.3 – 10.3[6] | 1.2 – 5.2[4,9,10] |
| POC:PN | 7.3– 10.2[6] | 5.8 – 9.0[4,9,10] |
| Chl a (μg $L^{-1}$) | 0.1 – 0.2[6] | 0.02 – 1.17[2] |
| Current velocity (cm $s^{-1}$) | 0 – 10[3] | 0 – 50[2,4,5] |
| Aragonite saturation ($\Omega_{arag}$) | 3.7 – 4.4[3] | 1.4 – 2.4[2] |
| pH | 8.2 – 8.3[3] | 7.92 – 8.19[2] |
| Salinity | 40.5 – 41.0[3] | 34.6 – 35.7[2] |

# Supplementary References


1 Fricke, H. W. & Schuhmacher, H. The depth limits of Red Sea stony corals: An ecophysiological problem (A deep diving survey by submersible). *Mar. Ecol.* **4**, 163-194, doi:10.1111/j.1439-0485.1983.tb00294.x (1983).

2 Findlay, H. S. *et al.* Fine-scale nutrient and carbonate system dynamics around cold-water coral reefs in the northeast Atlantic. *Scientific Reports* **4**, doi:10.1038/srep03671 (2014).

3 Silverman, J., Lazar, B. & Erez, J. Community metabolism of a coral reef exposed to naturally varying dissolved inorganic nutrient loads. *Biogeochemistry* **84**, 67-82, doi:10.1007/s10533-007-9075-5 (2007).

4 Wagner, H., Purser, A., Thomsen, L., Jesus, C. C. & Lundalv, T. Particulate organic matter fluxes and hydrodynamics at the Tisler cold-water coral reef. *J. Mar. Syst.* **85**, 19-29, doi:10.1016/j.jmarsys.2010.11.003 (2011).

5 Mienis, F. *et al.* Hydrodynamic controls on cold-water coral growth and carbonate-mound development at the SW and SE rockall trough margin, NE Atlantic ocean. *Deep-Sea*

*Research Part I-Oceanographic Research Papers* **54**, 1655-1674, doi:10.1016/j.dsr.2007.05.013 (2007).

6 Rix, L. *et al.* Seasonality in dinitrogen fixation and primary productivity by coral reef framework substrates from the northern Red Sea. *Mar. Ecol. Prog. Ser.* **533**, 79-92, doi:10.3354/meps11383 (2015).

7 Naumann, M. S. *et al.* Budget of coral-derived organic carbon in a fringing coral reef of the Gulf of Aqaba, Red Sea. *J. Mar. Syst.* **105**, 20-29, doi:10.1016/j.jmarsys.2012.05.007 (2012).

8 van Duyl, F. C., Hegeman, J., Hoogstraten, A. & Maier, C. Dissolved carbon fixation by sponge-microbe consortia of deep water coral mounds in the northeastern Atlantic Ocean. *Mar. Ecol. Prog. Ser.* **358**, 137-150, doi:10.3354/meps07370 (2008).

9 Kiriakoulakis, K., Freiwald, A., Fisher, E. & Wolff, G. A. Organic matter quality and supply to deep-water coral/mound systems of the NW European Continental Margin. *Int J Earth Sci* **96**, 159-170, doi:10.1007/s00531-006-0078-6 (2007).

10 Kiriakoulakis, K., Bett, B. J., White, M. & Wolff, G. A. Organic biogeochemistry of the Darwin Mounds, a deep-water coral ecosystem, of the NE Atlantic. *Deep-Sea Research Part I-Oceanographic Research Papers* **51**, 1937-1954, doi:10.1016/j.dsr.2004.07.010 (2004).
